# Genetic parameter estimation and genome-wide association study of fiber characteristics for cashmere goats in the United States

**DOI:** 10.1093/jas/skag073

**Published:** 2026-03-13

**Authors:** Elizabeth A Dressler, Jennifer M Bormann, Robert L Weaber, Roger C Merkel, Megan M Rolf

**Affiliations:** Department of Animal Sciences and Industry, Kansas State University, Manhattan, KS 66506, United States; Department of Animal Sciences and Industry, Kansas State University, Manhattan, KS 66506, United States; Department of Animal Sciences and Industry, Kansas State University, Manhattan, KS 66506, United States; American Institute for Goat Research, Langston University, Langston,OK 73050, United States; Department of Animal Sciences and Industry, Kansas State University, Manhattan, KS 66506, United States

**Keywords:** genetic correlation, genetic evaluation, heritability, repeatability, variance components

## Abstract

There are currently no genetic selection tools available to cashmere producers in the United States, presenting an opportunity for improvement of cashmere fiber characteristics through the development and implementation of such tools. Cashmere producers submitted sheared fiber samples to the Texas A&M Bill Sims Wool and Mohair Research Laboratory in San Angelo, Texas, for the measurement of mean fiber diameter (MFD), standard deviation of diameter (SDD), coefficient of variation of diameter (CVD), curvature (CURV), standard deviation of curvature (SDCurv), coarse edge micron (CEM), spinning fineness (SpinF), and percentage ≤19 µm (Perc_19). Staple length (SL) and greasy fleece weight (FW) were measured by individual producers using a ruler and scale, respectively. Pedigree records were provided by collaborating producers, and all goats were genotyped on the Goat GGP 70K BeadChip (Neogen Corp.). Variance components for each trait were estimated using the BLUPF90 suite of programs and a mixed animal model that included significant fixed effects: age at cashmere collection (year), sex, and contemporary group (farm x testing year). Estimated heritabilities for MFD, SDD, CVD, CURV, SDCurv, CEM, SpinF, Perc_19, SL, and FW were 0.49 ± 0.07, 0.27 ± 0.07, 0.29 ± 0.07, 0.48 ± 0.06, 0.38 ± 0.07, 0.36 ± 0.07, 0.45 ± 0.07, 0.50 ± 0.07, 0.48 ± 0.07, and 0.52 ± 0.19, respectively. In general, repeatability of cashmere fiber traits were moderate to high. Both phenotypic and genetic correlations varied between pairs of traits. The genetic correlations MFD had with CVD, SDCURV, SL, and FW were unfavorable and warrant consideration to avoid antagonistic correlated responses to selection. Significant single nucleotide polymorphisms (SNPs) were identified for cashmere fiber characteristics. Several genes located within the QTL region (± 50 kb) of these SNPs have previously been associated with fiber production or related biological processes. These results suggest that cashmere fiber traits are heritable, and genetic improvement of cashmere fiber production is feasible. This study could provide a foundation for the future development of a genetic selection tool for U.S. cashmere producers.

## Teaser

This study is a novel genetic evaluation of fiber characteristics from cashmere goats in the United States. Heritability, repeatability, phenotypic and genetic correlations, and significant SNPs are reported.

## Introduction

Cashmere, one of the finest and softest animal fibers (McGregor 2014), is the down produced from the secondary hair follicles of some goats. Cashmere-type goats originated throughout the Himalayan region, and China and Mongolia currently produce approximately 85% of the world’s cashmere (Van Der Westhuysen 2005). However, cashmere production also occurs in other countries, including Australia, New Zealand, the United Kingdom, and the United States. The cashmere industry in the United States is relatively new, but it is an important commodity to small stakeholders. Production occurs across the entire U.S., predominantly from relatively small operations, rather than large-scale commercial enterprises. Many breeds of goats can grow down fiber. However, only fiber that meets certain standards can be marketed as cashmere; primarily, fiber must not exceed a mean fiber diameter (MFD) of 19 µm in the United States (FTC 1939).

Fiber characteristics such as MFD, measures of diameter variation such as standard deviation (SDD) and coefficient of variation (CVD), curvature (CURV), length and weight are important in cashmere production. The genetic parameters associated with these economically relevant fiber phenotypes and others have been studied in populations across the world including China, New Zealand, the United Kingdom, Australia, and Kyrgyzstan. Estimated heritabilities of these fiber traits vary widely across studies, both within and between countries (as reviewed by Dressler et al 2024). For example, the estimated heritability of MFD in Australian studies ranged from 0.39 ± 0.16 to 0.70 ± 0.19 (Couchman and Wilkinson 1987; Pattie and Restall 1989; Gifford et al 1990; Rose et al 1992). In contrast, the heritability of MFD reported in studies from China ranged from 0.14 to 0.41 ± 0.08 (Jinquan et al 2001; Zhou et al 2002; Bai et al 2005; Wang et al 2015; Pallotti et al 2018). Heritability estimates may differ for a variety of reasons, such as differences in selection history, population structure, breed, statistical modeling, or trait measurement technique.

Although cashmere fiber traits have been evaluated in other countries, no genetic analyses have been published in the United States. Further, the United States lacks a national genetic evaluation program for cashmere traits, which remains a key challenge for U.S. cashmere producers. The only selection tools U.S. cashmere producers currently have are phenotypic selection and/or selection with pedigree information (Rupp et al 2016). This has hindered the breeding progress possible for producers (Shumbusho et al 2013; Shumbusho et al 2016). Thus, U.S. cashmere producers would greatly benefit the development of a national genetic evaluation program for cashmere fiber traits. Establishing such a program requires foundational research on genetic parameters in U.S. cashmere populations.

The objective of this study was to estimate the heritability of economically relevant cashmere fiber traits, estimate the phenotypic and genetic correlations between fiber traits, and perform a genome-wide association study (GWAS) to identify regions of the genome associated with fiber traits for cashmere goats in the United States.

## Materials and methods

### Study population and design

Animal procedures on Langston University goats were performed as a part of Langston University Institutional Animal Care and Use project number OKLUMERKEL2020, experiment number RM-22-03, in accordance with Federation of Animal Science Societies guidelines (FASS 2010). Data from other U.S. cashmere goats were collected by producers with normal animal management processes, therefore, the Animal Care and Use Committee approval was not required.

**Figure 1:**
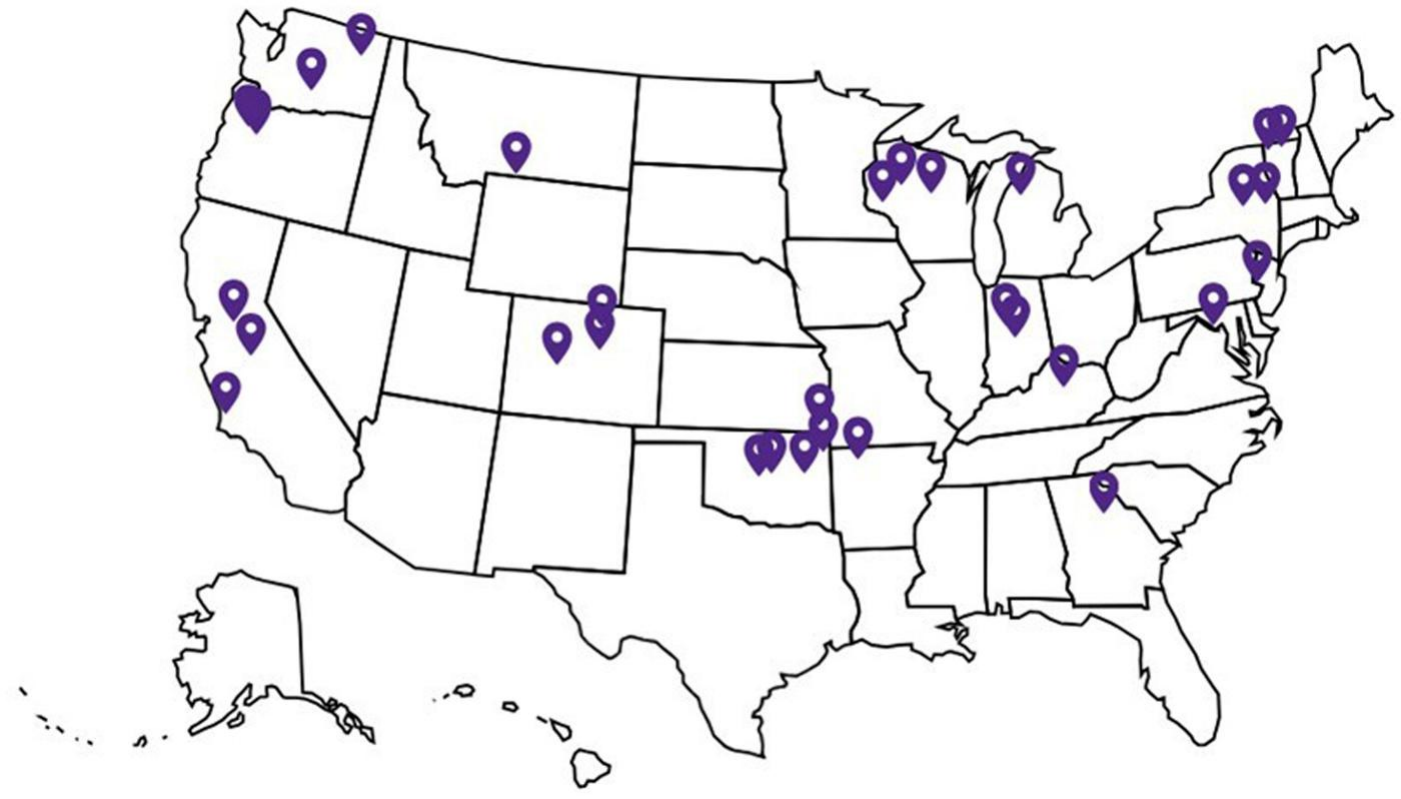
Map of 32 producer locations across the United States.

Thirty-two cashmere producers located across the continental United States voluntarily contributed data to this study. Cashmere flocks that participated in this study were located in 17 different states (Table 1; Figure 1). In exchange for participation in this study, participants received the genotype and fiber testing results for animals in their flock at no cost. A total of 1,248 records from 1,040 cashmere goats were available. All sexes were used in the study: whether (191 animals, 242 records), doe (720 animals, 845 records), and buck (128 animals, 160 records). The age at cashmere collection ranged from 0.17 yr (∼2 mo) to 18.7 yr, but the average was 2.72 yr and median was 1.83 yr.

**Table 1:** Cashmere producer information.

Farm number	Location	Number of records	Number of animals
**01**	OK	182	182
**02**	OK	541	391
**03**	CA	58	30
**04**	OR	14	13
**05**	NY	9	9
**06**	WA	20	20
**07**	MI	13	13
**08**	KS	80	73
**09**	VT	7	7
**10**	AR	3	3
**11**	CO	46	32
**12**	VT	16	16
**13**	IN	15	15
**14**	NJ	8	8
**15**	IN	18	18
**16**	OR	37	33
**17**	WI	49	49
**18**	OK	4	4
**19**	KY	2	2
**20**	NY	6	6
**21**	CA	26	22
**22**	CO	5	5
**23**	CO	14	14
**24**	GA	8	8
**25**	MD	4	4
**26**	WI	6	6
**27**	WI	31	31
**28**	MT	10	10
**29**	OR	4	4
**30**	OK	4	4
**31**	CA	1	1
**32**	WA	7	7
**Total**		1,248	1,040

### Fiber test

Collaborating producers collected fiber samples between December 2021 and May 2024 during cashmere harvest. Fiber samples were collected by clipping a 2 inch × 2 inch fiber sample from the mid-side (McGregor 1994) of each goat. Mid-side samples are the best single predictor of overall fleece characteristics (Couchman and McGregor 1983). A mid-side sample is clipped above the last rib with standard hair clippers. A size 30 clipping blade or similar was used to retain the full length of the clip. Producers individually packaged and labeled cashmere samples for submission to the testing laboratory. During sample collection, some producers measured staple length (SL; mm) and total fleece weight (FW; g). Staple length was measured on the clipped, unstretched fiber sample using a ruler. Fleece weight was measured using a scale for the entire greasy fleece after harvest, with nearly all fleeces collected by shearing and a small number (n = 22) by hand combing.

Fiber samples were sent to the Texas A&M Bill Sims Wool and Mohair Research Laboratory in San Angelo, Texas. Analysis of the cashmere fiber was done with an Optical Fibre Diameter Analyser 100 (OFDA 100; Baxter et al 1992). The OFDA 100 was introduced in the early 1990’s to analyze fiber characteristics of wool, but the instrument can also be used to evaluate cashmere fiber. Samples were processed upon arrival at the laboratory in preparation for testing with the OFDA 100, including “guillotining” samples into short ∼2mm snippets, washing and drying samples to remove grease, dirt, and other impurities, and arranging snippets on a slide for analysis.

The fiber characteristics analyzed by the OFDA 100 were MFD, SDD, CVD, coarse edge micron (CEM), CURV, standard deviation of curvature (SDCurv), and spinning fineness (SpinF). A frequency histogram was also generated by the OFDA 100 for each sample. The frequency histogram specifies the total number of fibers present in the sample at each 1 µm diameter interval from 4 µm to 27 µm (12/5/2023 to 01/25/2024; 68 records) or 4 µm to 30 µm (all other records). Using the diameter frequency histogram data, the trait Percentage ≤ 19 µm (Perc_19) was calculated. Cashmere fiber trait definitions are in Table 2.

**Table 2:** Definitions and abbreviations of cashmere fiber characteristics.

Trait	Abbreviation	Definition
**Mean fiber diameter, µm**	MFD	Average fiber diameter of fibers in the tested sample ≤30 µm
**Standard deviation of diameter, µm**	SDD	Standard deviation of fiber diameter, calculated using the population formula.
**Coefficient of variation of diameter, %**	CVD	Coefficient of fiber diameter calculated by: $(\frac{SD\;\mathrm{Mean}}{\mathrm{MFD}})*100$
**Course edge micron, µm**	CEM	The number of micron above the average diameter where the coarsest 5% of fibers lie
**Curvature, °/mm**	CURV	The average fiber curvature
**Standard deviation of curvature, °/mm**	SDCurv	Standard deviation of fiber curvature, calculated using the population formula.
**Spinning fineness, µm**	SpinF	A measure of the performance of the fiber when spun into yarn calculated from CVD and MFD. Original theory proposed by Martindale (1945) and formula used proposed by Butler and Dolling (1992).
**Staple length, mm**	SL	Length of clipped fiber sample
**Fleece weight, g**	FW	Total fleece weight after harvest
**Percentage ≤19, %**	Perc_19	Percentage of fibers with diameters between 4 and 30 µm that are less than or equal to 19 µm

### Genotype information

Collaborating cashmere producers provided DNA samples for genotyping via a hair sample. Producers were encouraged to collect the hair samples at the same time as the mid-side fiber sample was clipped. Hair samples for genotyping were collected by the producer from each goat by plucking ∼20 guard hairs from the tail or spinal ridge, ensuring that the follicle was still attached at the base. Hair samples were sent to Neogen Corporation (Lincoln, NE) for DNA extraction and genotyping. DNA samples were genotyped on the Goat GGP 70K BeadChip (Version 1; Neogen Corp.). A total of 1,462 genotypes were available for 1,036 U.S. animals and 426 related New Zealand animals. Phenotypic, pedigree, and genotypic data were available for U.S. animals, whereas only genotypes from related New Zealand animals were utilized to inform the estimation of genetic relationships. Genotypes from animals with a call rate less than 0.90 were removed, leaving 1,372 genotyped animals for analysis. Genotypes were further filtered for quality control by removing 4,393 single nucleotide polymorphisms (SNPs) with a minor allele frequency less than 0.05 and 9,449 SNPs with a SNP call rate less than 0.90. The number of SNPs remaining for analysis was 54,363.

### Pedigree information

Available pedigree information was provided by collaborating cashmere producers. The depth of the provided pedigree was variable but included all available pedigree information. The number of generations in the pedigree ranged from 1 to 5 and the final pedigree included 1,910 animals from 26 farms. Pedigree connectedness among farms was primarily through shared male germplasm, with 24 sires used on more than one farm.

### Statistical analysis

Summary statistics including minimum, mean, maximum, and standard deviation were calculated in R Studio. Phenotypic correlations between fiber characteristics were calculated in SAS v9.4 using the PROC CORR function.

Established scientific literature indicates factors such as age at sampling, birth type, rearing type, liveweight at sampling, and sex influence cashmere fiber production (James 2009; Wang et al 2019). All available factors were extracted from the data and used in model selection for each fiber trait. Model fit was assessed with the Akaike information criterion (AIC). Forward stepwise selection was employed, and additional variables were retained only if their inclusion resulted in meaningful improvement in model fit, defined as a reduction in AIC of at least three units.

The base model for each trait was as follows:


$$y_{i}=\;b_{0}+\;e_{i}$$

where $y_{i}$ is the fiber trait being evaluated, $b_{0}$ is the intercept, and $e_{i}$ is the residual. The following fixed effects were tested with forward selection: sex (doe, buck, wether), age at cashmere collection (years), liveweight (kg), and contemporary group (farm x fiber testing year). Liveweight was only reported by a portion of participating cashmere producers, resulting in only 631 out of 1,243 records with a liveweight observation. For most traits, the inclusion of liveweight resulted in model improvement (Dressler 2025). However, due to the substantial reduction in sample size from including liveweight as a fixed effect in the current dataset, final models did not include liveweight. Liveweight is likely an important effect for modeling cashmere traits, and in the future, particularly for the purposes of a national genetic evaluation, more cashmere producers should consider weighing their goats at cashmere harvest. Final models for MFD, CVD, and SDCurv included the fixed effects of contemporary group, age at collection, and sex. Contemporary group and age at collection were the fixed effects included in the final model for SDD, CEM, SpinF, and Perc_19. Final models for CURV and SL included contemporary group and sex. Sex was the only significant fixed effect included in the model for FW.

### Genetic parameter estimation

For all traits, variance components were estimated using average information restricted maximum likelihood in the BLUPF90 suite programs (Misztal et al 2014). Single-step genomic BLUP was used where the numerator relationship matrix, **A**^−1^ is replaced by **H**^−1^ (Aguilar et al 2010) as follows:


$$H^{-1}=\;A^{-1}+\;[\begin{array}{cc} 0 & 0\\ 0 & G^{-1}-\;A_{22}^{-1} \end{array}]$$

where **H** is the relationship coefficients matrix between animals, **A** is the additive relationship matrix, **A**_22_ is the numerator relationship matrix for genotyped animals, and **G** is the genomic relationship matrix (VanRaden 2008) as **G= ZZ’**, where:


$$Z=\;\frac{M-P}{{\left[2\Sigma p_{j}(1-p_{j})\right]}^{1/2}}$$

where **Z** is the centered genotype matrix, **M** is the matrix of marker genotypes for each individual (coded as 0, 1, 2 copies of the second allele), and **P** is the matrix of 2p_j_ (frequency of second allele, p, at locus *j*).

First, a univariate single record analysis was performed with only the first record available from each animal included in the analysis. The general univariate animal models were:


y=Xb+Zu+e

where y is a vector of dependent variables, b is a vector of fixed effects selected from forward selection, u is a vector of direct additive genetic effects, e is a vector of random residuals and **X** and **Z** are incidence matrices for b and u, respectively.

Next, bivariate animal models were run with each pairwise combination of fiber traits to obtain genetic correlations. Then, a repeated records analysis was run for each trait which included every fiber record available using the following animal model:


y=Xb+Zu+Wp+e

where elements are the same as the previous animal model with the addition of **W**, the incidence matrix relating p to y, and p which is a vector of permanent environmental effects.

### Genome-wide association study

For each trait, the SNP effects were obtained from the single record univariate analysis. The postGSf90 program within the BLUPF90 program suite (Misztal et al 2014) was used to estimate SNP effects, prediction error variance, and *P*-values. A single-step GWAS was done to predict SNP effects as follows (Wang et al 2012):


a=Zu

where a is a vector of breeding values for genotyped animals, Z is a matrix relating genotypes of each locus, and u is a vector of SNP effects. The variances are as follows:


$$\mathrm{var}(a)=\mathrm{var}\;(Zu)=G\sigma_{a}^{2}=\mathrm{ZIZ}\prime \lambda$$

where I is an identity matrix, λ is the variance ratio for SNP effects and breeding values, and a, u, **G**, and **Z** were defined previously (Masuda 2025).

The prediction of SNP effects is as follows:


$$\hat{u}=\mathrm{IZ}\prime {\left(\mathrm{ZIZ}\prime \right)}^{-1}\hat{a}$$

where $\hat{u}$ is a vector of SNP effects, $\hat{a}$ is a vector of breeding values, and **I** and **Z** were defined previously (Masuda 2025).

False discovery rate was calculated for each SNP using the q-value package in R (Storey et al 2025) and no SNPs were significant at a threshold of <0.05. A suggestive significance threshold of -log_10_  *P*-value ≥4 was set for identifying SNPs of interest. Manhattan plots of the SNP effects were created using the qqman package in R (Turner 2018).

Quantitative trait loci (QTL) regions of ± 50 kb were formed around significant SNP positions to account for linkage disequilibrium in the caprine genome (Brito et al 2015). The GALLO package in R (Fonseca et al 2020) and the National Center for Biotechnology Information Capra hircus GFF annotation file for the USDA ARS 1.2 genome assembly (Bickhart et al 2017; accession GCF_001704415.2) was used to identify genes within each QTL region. The UniProt Consortium (2023) was used to identify molecular and biological gene functions. The QTL regions were compared with previously reported trait associations in the Goat QTLdb (Hu et al 2022). Because there are far fewer genomic studies in goats compared to other livestock species, gene candidates were searched in the Sheep QTLdb to identify previous associations with traits in sheep.

## Results and discussion

### Summary statistics

Summary statistics for fiber trait phenotypes are presented in Table 3. The average MFD was 15.34 µm for this population of goats, which is below the maximum textile standard threshold for fiber to be considered cashmere, which is 19 µm. The average MFD in this study is very similar to the average reported by Pattie and Restall (1989) for an Australian feral, unselected population of goats but greater than a mean MFD of 13.59 µm from a population of Inner Mongolia White Cashmere goats (Bai et al 2005).

**Table 3:** Summary statistics of animal information and fiber traits for all records.

Trait	n	Mean	Minimum	Maximum	Standard deviation
**Age at collection, yr**	1,080	2.72	0.17	18.74	2.87
**Mean fiber diameter, µm**	1,079	15.34	11.30	22.52	1.76
**Standard deviation of diameter, µm**	1,080	3.17	2.03	6.92	0.52
**Coefficient of variation of diameter, %**	1,079	20.67	15.20	41.80	2.86
**Coarse edge micron, µm**	1,080	6.16	3.07	14.6	1.22
**Curvature, °/mm**	1,242	67.76	21.30	123.80	11.87
**Standard deviation of curvature, °/mm**	1,079	40.81	17.00	75.20	8.43
**Spinning fineness, µm**	1,080	14.90	11.01	21.76	1.70
**Staple length, mm**	987	60.34	4.30	120.0	21.57
**Fleece weight, g**	260	237.8	20.0	644.0	94.11
**Percentage less than 19 µm, %**	1,080	87.51	27.7	99.7	11.52

Course edge micron, SpinF, and Perc_19 have not been widely studied and currently lack information in the literature to make comparisons. However, SDD, CVD, CURV, and SDCURV had averages in the current study that were generally similar to literature values. Staple length had an average of 60.34 mm, which is substantially shorter than lengths reported in other studies. Average staple length of sheared cashmere goats across 11 farms in Australia was 87 mm (McGregor and Butler 2008b). Combed Inner Mongolia cashmere goats had an average staple length of 168.6 mm across 11,854 records (Bai et al 2005). The method used to harvest cashmere can influence staple length, with combing generally producing longer staples compared to shearing, which could be one reason average SL is shorter in this study compared to others.

The average greasy FW was 237.8 g, however, only 260 records had a FW recorded. Female and male progeny of feral Australian goats had average FW of 287 and 338 g, respectively (Restall and Pattie 1989). More recently, average greasy FW of Australian cashmere goats across 11 farms was reported as 394 g, which suggests that improvement in FW has been made in Australian populations (McGregor and Butler 2008b). The average FW of these U.S. goats is most similar to the primarily unselected feral Australian goats from the 1980’s, indicating substantial potential for genetic improvement in U.S. populations.

### Heritability and repeatability of cashmere fiber traits

Single record heritability estimates from the univariate analysis are on the diagonal of Table 4 and from each bivariate analysis in Table 5. For all traits, the heritability estimate from the single-record univariate analysis was very similar to the average heritability of single-record bivariate analyses. Heritability and repeatability estimate from the repeated records analysis are in Table 6. In general, heritability estimates were similar between the single record and repeated records analyses for each trait, except for FW. The heritability estimates for FW differed considerably between the univariate single record and repeated records analyses, which may be due to the very small number of FW records available in the single record analysis.

**Table 4:** Estimates from the single record analysis of cashmere fiber traits: univariate heritability estimates (bold diagonal), phenotypic correlations (upper triangle), and genetic correlations ± standard error (lower triangle) from each bivariate analysis.

	MFD	SDD	CVD	CEM	CURV	SDCurv	SpinF	SL	FW	Perc_19
**MFD, µm**	**0.49 ± 0.07**	0.60**	−0.13**	0.52**	−0.70**	−0.49**	0.93**	0.06	0.38**	−0.94**
**SDD, µm**	0.52 ± 0.14	**0.27 ± 0.07**	0.70**	0.91**	−0.41**	−0.18**	0.73**	−0.07*	−0.01	−0.70**
**CVD, %**	−0.32 ± 0.16	0.63 ± 0.15	**0.29 ± 0.07**	0.66**	0.12**	0.22**	0.08*	−0.15**	−0.34**	−0.04
**CEM, µm**	0.48 ± 0.13	0.95 ± 0.03	0.47 ± 0.15	**0.36 ± 0.07**	−0.33**	−0.12**	0.65**	−0.02	0.07	−0.60**
**CURV, °/mm**	−0.74 ± 0.06	−0.42 ± 0.16	0.21 ± 0.17	−0.38 ± 0.14	**0.48 ± 0.06**	0.70**	−0.66**	−0.16**	−0.20*	0.64**
**SDCurv, °/mm**	−0.59 ± 0.10	−0.22 ± 0.19	0.27 ± 0.18	−0.14 ± 0.17	0.78 ± 0.07	**0.38 ± 0.07**	−0.42**	−0.19**	−0.18*	0.42**
**SpinF, µm**	0.98 ± 0.01	0.68 ± 0.10	−0.15 ± 0.18	0.63 ± 0.10	−0.71 ± 0.07	−0.55 ± 0.12	**0.45 ± 0.07**	0.03	0.32**	−0.92**
**SL, mm**	0.34 ± 0.13	0.30 ± 0.18	0.08 ± 0.18	0.30 ± 0.16	−0.57 ± 0.11	−0.67 ± 0.12	0.35 ± 0.14	**0.48 ± 0.07**	0.55**	−0.04
**FW, g**	0.40 ± 0.22	0.05 ± 0.58	−0.39 ± 0.61	0.08 ± 0.46	−0.48 ± 0.25	−0.56 ± 0.27	0.37 ± 0.24	0.66 ± 0.25	**0.52 ± 0.19**	−0.29**
**Perc_19, %**	−0.95 ± 0.02	−0.58 ± 0.11	0.25 ± 0.17	−0.52 ± 0.11	0.71 ± 0.08	0.55 ± 0.12	−0.96 ± 0.01	−0.31 ± 0.13	−0.62 ± 0.21	**0.50 ± 0.07**

*Phenotypic correlations significantly different from zero at *P *< 0.05.

**Phenotypic correlations significantly different from zero at *P *< 0.0001.

**Table 5:** Heritability estimates (with SE below) of traits listed in the first column from single record bivariate analysis with all other traits.

Trait:	Heritabilities from bivariate analyses with all other traits									
	MFD	SDD	CVD	CEM	CURV	SDCurv	SpinF	SL	FW	Perc_19
**MFD, µm**	**0.497 ** **0.06-0.07**	0.50 0.07	0.50 0.06	0.49 0.07	0.50 0.06	0.50 0.06	0.49 0.07	0.49 0.06	0.50 0.06	0.50 0.06
**SDD, µm**	0.27 0.07	**0.274 ** **0.07**	0.27 0.07	0.29 0.07	0.27 0.07	0.28 0.07	0.27 0.07	0.28 0.07	0.27 0.07	0.27 0.07
**CVD, %**	0.29 0.07	0.29 0.07	**0.289 ** **0.07**	0.29 0.07	0.29 0.07	0.29 0.07	0.29 0.07	0.29 0.07	0.29 0.07	0.30 0.07
**CEM, µm**	0.36 0.07	0.37 0.07	0.38 0.07	**0.367 ** **0.07**	0.37 0.07	0.37 0.07	0.36 0.07	0.37 0.07	0.37 0.07	0.36 0.07
**CURV, °/mm**	0.45 0.07	0.45 0.07	0.45 0.07	0.45 0.07	**0.458 ** **0.06-0.07**	0.46 0.07	0.45 0.07	0.47 0.06	0.48 0.06	0.45 0.07
**SDCurv, °/mm**	0.38 0.07	0.38 0.07	0.38 0.07	0.38 0.07	0.39 0.07	**0.381 ** **0.07**	0.38 0.07	0.37 0.07	0.38 0.07	0.38 0.07
**SpinF, µm**	0.44 0.07	0.45 0.07	0.45 0.07	0.45 0.07	0.45 0.07	0.45 0.07	**0.451 ** **0.07**	0.45 0.07	0.45 0.07	0.47 0.07
**SL, mm**	0.45 0.07	0.45 0.07	0.45 0.07	0.45 0.07	0.49 0.07	0.47 0.07	0.45 0.07	**0.457 ** **0.07**	0.46 0.07	0.45 0.07
**FW, g**	0.55 0.16	0.29 0.18	0.38 0.18	0.39 0.19	0.62 0.17	0.59 0.17	0.58 0.17	0.49 0.15	**0.506 ** **0.15-0.19**	0.65 0.16
**Perc_19, %**	0.50 0.07	0.51 0.07	0.51 0.07	0.50 0.07	0.50 0.07	0.50 0.07	0.50 0.07	0.49 0.07	0.50 0.07	**0.501 ** **0.07**

Average heritability from all bivariate analyses for a trait is bolded on the diagonal.

**Table 6:** Heritability, repeatability, and variance components for repeated records of cashmere fiber traits.

Trait	Additive genetic variance	Residual variance	Permanent environmental variance	Heritability ± standard error	Repeatability ± standard error
**Mean fiber diameter, µm**	1.0777	0.47348	0.44715	0.54 ± 0.06	0.76 ± 0.03
**Standard deviation of diameter, µm**	0.05677	0.05251	0.09205	0.28 ± 0.07	0.74 ± 0.04
**Coefficient of variation of diameter, %**	2.3138	1.7794	3.0379	0.32 ± 0.06	0.75 ± 0.04
**Coarse edge micron, µm**	0.4658	0.2436	0.5591	0.37 ± 0.07	0.81 ± 0.03
**Curvature °/mm**	59.531	38.201	14.377	0.53 ± 0.06	0.66 ± 0.04
**Standard deviation of curvature, °/mm**	13.035	17.162	4.9845	0.37 ± 0.07	0.51 ± 0.07
**Spinning fineness, µm**	0.8919	0.4436	0.5125	0.48 ± 0.07	0.76 ± 0.03
**Staple length, mm**	124.07	141.54	15.676	0.44 ± 0.06	0.49 ± 0.07
**Fleece weight, g**	1834.70	6374.0	0.0023	0.22 ± 0.11	0.22 ± 0.11
**Percentage less than 19 µm, %**	49.703	27.129	16.35	0.53 ± 0.06	0.71 ± 0.04

The univariate single record and repeated records heritabilities for MFD were 0.49 ± 0.07 and 0.54 ± 0.06, respectively. The repeatability was 0.76 ± 0.03. The heritability of MFD is generally moderate to highly heritable in literature, despite many factors that could influence estimates. Pattie and Restall (1989) reported a heritability and repeatability for MFD of 0.47 ± 0.15 and 0.60 ± 0.02, respectively. This heritability estimate is similar to the current study, while the repeatability is slightly lower. There were differences in population and fiber analysis machinery between this study and the current study, which could contribute to the slight differences. In China, Pallotti et al (2018) reported a similar MFD heritability of 0.41 ± 0.08. Lower heritabilities were reported in other Chinese studies of 0.36 ± 0.03, 0.32, and 0.28 (Zhou et al 2002; Bai et al 2005; Rong et al 2024). Differences in animal age at collection and cashmere collection method could be reasons for differences in estimates.

Measures of fiber diameter uniformity (SDD and CVD) had moderate univariate heritabilities in both the single record and repeated records analyses (Tables 4 and 6). Repeatability of SDD and CVD was high (Table 6). The heritability of SDD for 5-mo-old kids in two studies were 0.43 ± 0.08 and 0.28 (Bishop and Russel 1996; Bishop and Allai 2000). 10-month-old Australian cashmere goats had a heritability of 0.38 ± 0.14 for SDD (Gifford et al 1990). These estimates are similar to those reported in the current study for SDD, especially considering the standard errors associated with the estimates. Compared to SDD, there is less literature available about CVD. Pallotti et al (2018) reported a higher heritability for CVD (0.52 ± 0.06) compared to the current study.

Coarse edge micron had similar moderate heritability estimates in both the single and repeated records analyses and high repeatability (Tables 4 and 6). Several different descriptors of the coarse edge of the fiber diameter distribution have been described (Whiteley and Thompson 1985). In the current study, CEM is defined as the number of microns above the MFD at which the coarsest 5% of fibers begin. For example, the average MFD in the dataset is 15.34 µm and the average CEM is 6.16 µm, meaning that on average the coarsest 5% of fibers begin at 21.5 µm. Lower CEM values indicate a more consistent, finer fiber, and higher values indicate a broader distribution of fiber diameter with more coarse fibers. Others have defined coarse edge as the percentage of fibers with diameters above a threshold, usually 30 or 35 µm. There are no heritability estimates in the literature for cashmere using the same CEM definition to compare results.

Heritability and repeatability of CURV were high (Tables 4 and 6). Style refers to the subjective assessment of fiber crimp characteristics, with curvature being a key contributing factor (McGregor 2007). The fiber style category of Faure Island cashmere goats at 10 and 17 mo of age had heritabilities of 0.27 ± 0.11 and 0.38 ± 0.12, respectively (McGregor 1997). This is lower than the current study, but differences could be expected because these are not the exact same traits. Heritability estimates in literature for cashmere fiber CURV are limited; however, our estimates are similar to CURV heritabilities measured in other species. A study of alpacas with extremely fine fiber (average MFD= 16.67 µm) in the U.S. reported a heritability for fiber CURV of 0.50 (Wuliji 2017). In Merino sheep, similar heritability (0.39 ± 0.04) and repeatability (0.64 ± 0.02) estimates for CURV were reported by Mortimer et al (2017) and Hatcher and Atkins (2000), respectively.

In both the single and repeated records analysis, the univariate heritability of SDCurv was moderate (Tables 4 and 6). The repeatability of SDCurv was 0.51 ± 0.07, which is high, but not as high as the other fiber characteristics in this study (Table 6). Interestingly, SDCurv was less heritable than CURV. The range in fixed-effect solutions for the contemporary group (farm x year) was substantial (26.35 to 61.40) with a standard deviation of 7.85, indicating considerable variation in SDCurv across contemporary groups. This supports the findings of McGregor and Butler (2010), who reported that SDCurv varied largely by farm and accounted for 41% of the phenotypic variation in SDCurv. As with CURV, literature on genetic parameters associated with SDCurv is scarce, so comparisons of heritability and repeatability estimates are not possible.

The single record and repeated records univariate heritabilities of SpinF were 0.45 ± 0.07 and 0.48 ± 0.07, respectively. The repeatability of SpinF was 0.76 ± 0.03. In their foundational paper on SpinF, Butler and Dolling (1992) proposed an approach to approximate the heritability of SpinF using the phenotypic and genetic parameters of MFD and CVD, rather than SpinF itself. Derived heritabilities were 0.43, 0.56, and 0.70, varying with the application of the methodology to different sets of previously published estimates (Butler and Dolling 1992). Two of these estimates are greater than the heritability in the current study, which could be because they were derived from other parameters instead of directly estimated.

The univariate heritability of SL in both the single record and repeated records analysis was moderately high (Tables 4 and 6). In contrast to most other traits where repeatability was much higher than the heritability, the repeatability for SL was similar to the heritability (Table 6). Inner Mongolia cashmere goats had heritability estimates for SL of 0.32 and 0.29 (Jinquan et al 2001 and Bai et al 2005, respectively). However, SL measurement methods used in these two studies are unclear, which may contribute to differences in estimates compared to the current study. Additionally, Inner Mongolia cashmere goats have experienced different selection and management than the US goats in this study. Down fiber length is more commonly reported in cashmere studies and is generally moderately to highly heritable, though there is a wide range of published estimates (as reviewed by Dressler et al 2024). These estimates are not directly comparable to the current study because down fiber length refers to the length of only the fine undercoat fibers, typically after dehairing. In contrast, staple length is the length of an entire lock or cluster of fibers and reflects a more general fleece trait measured prior to processing.

The univariate heritability of FW was high at 0.52 ± 0.19 for the single record analysis. However, the heritability and repeatability of FW was much lower at 0.22 ± 0.11 and 0.22 ± 0.11, respectively, in the repeated records analysis. Permanent environmental variance was very small (Table 6), resulting in heritability and repeatability estimates that are approximately the same in the repeated records analysis. The FW reported in this study is a greasy FW, which was weighed immediately after harvest without any dehairing or cleaning of containments. Since greasy FW includes variable amounts of environmental material such as dirt and vegetable matter, this may cause greater phenotypic variance and, as a result, lower heritability and repeatability estimates, as observed in the repeated records analysis. Bigham et al (1993) reported a greasy FW heritability of 0.42 ± 0.13 for yearling goats in New Zealand. Heritability of greasy FW was reported as 0.42 ± 0.16 and 0.67 ± 0.22 in two studies of 10-month-old Australian goats (Gifford et al 1990 and Rose et al 1992, respectively). These three previously published FW heritability estimates were from single record analyses and align better with the estimate from the single record analysis in the current study. The repeatability of greasy FW in Australian feral goats was 0.68 ± 0.02 (Pattie and Restall 1989), which is much higher than the current study. In this study, only one farm provided FW data, with 177 records used in the single-record analysis and 260 records in the repeated records analysis. The additional data provided from the repeated records provided more data for parameter estimation and may have better captured the environmental noise associated with greasy FW. The standard errors associated with FW estimates are larger than other traits where more data was available, as expected. Due to these factors, the FW estimates in this study should be viewed with caution and need to be validated in a subsequent study with a larger sample size and data from a more diverse array of producers.

Heritability and repeatability of Perc_19 were high (Tables 4 and 6). The most notable standard for cashmere is a threshold for MFD of ≤ 19 µm, which is how the textile industry defines and markets cashmere. This novel phenotype characterizes the proportion of down fibers (≤ 30 µm) within an individual fiber sample that meets this criterion. Selection for Perc_19 may be valuable because increasing the percentage of fine down fibers would help lower the MFD and reduce the number of fibers on the coarse edge. However, selection for both Perc_19 and MFD is likely unnecessary, and efforts to reduce fiber diameter could be focused on either MFD or Perc_19. Because Perc_19 is a novel trait, there are no heritability or repeatability estimates available in the literature for comparison.

### Genetic and phenotypic correlations between cashmere fiber traits

#### Correlations between MFD and other fiber characteristics

Phenotypic correlations between fiber traits reported in this study are shown in the upper diagonal portion of Table 4, and genetic correlations are in the lower diagonal of Table 4 from single-record bivariate analyses. Genetic covariances between fiber characteristics are presented in Table S1. Selection for decreased MFD, or at least maintaining MFD, is a common breeding objective. However, the correlations MFD has with other fiber characteristics are important considerations so that selection focused on MFD does not lead to unacceptable quality of other fiber characteristics. MFD had moderate to strong phenotypic and genetic correlations with most other fiber traits. The phenotypic and genetic correlations between MFD and SDD were 0.60 and 0.52 ± 0.14, respectively. This strong positive correlation is favorable because it suggests that selection to decrease MFD would result in decreased SDD, or less variability. Similar phenotypic and genetic correlations between MFD and SDD of 0.50 and 0.68 ± 0.09 were reported for cashmere goats in the United Kingdom. (Bishop and Russel 1996). A slightly lower phenotypic correlation of 0.41 and a higher genetic correlation of 0.79 ± 0.09 was reported by Bigham et al (1993).

Interestingly, the phenotypic and genetic correlations between MFD and CVD are negative, at −0.13 and −0.32 ± 0.16, respectively. Given the standard error, the genetic correlation may be lower; however, selection for decreased MFD could result in increased CVD. This difference could be because SDD is a direct measure of fiber diameter uniformity, whereas CVD expresses variability relative to MFD. If MFD decreases but SDD does not decrease proportionally, CVD increases. Thus, the negative correlation suggests that finer fleeces tend to have greater variability when fiber diameter is expressed as a proportion of their mean. This result is not in agreement with a study of Alashan Left Banner White cashmere goats, which reported positive phenotypic and genetic correlations between MFD and CVD of 0.29 ± 0.07 and 0.39 ± 0.10 (Pallotti et al 2018). This is more consistent with the correlation between MFD and SDD in the current study, where variability decreases with smaller diameter.

The phenotypic and genetic correlations between MFD and CEM were positive and favorable, at 0.52 and 0.48 ± 0.13, respectively. These correlations indicate that a higher MFD tends to be associated with a greater CEM, or the spread between the MFD and the coarsest 5% of fibers, indicating more down fibers on the coarse end of the distribution. Alternatively, selection for finer MFD would have an advantageous effect on CEM, resulting in improved fiber quality and handle, or the subjective feel of softness.

Both the phenotypic and genetic correlations between MFD and CURV were strongly negative, at −0.70 and −0.74 ± 0.06, respectively. This suggests that fiber with finer MFD will have greater curvature, which is likely a favorable correlation. Greater curvature is associated with more efficient processing (McGregor and Butler 2008a). Additionally, North American cashmere goat breed standards require fiber to have curvature greater than 45°/mm. In a study of Australian cashmere goats, increased MFD was strongly associated with a decline in fiber curvature and MFD accounted for 39% of the variation in fiber curvature (McGregor and Butler 2009). MFD and SDCurv had negative phenotypic and genetic correlations, −0.49 and −0.59 ± 0.10, respectively. These correlations suggest that selection for decreased MFD would also lead to increased CURV and SDCurv.

The phenotypic and genetic correlations between MFD and SpinF were very strong (0.93 and 0.98 ± 0.01, respectively). SpinF is calculated with an equation that includes MFD and CVD (Butler and Dolling 1992); therefore, a strong correlation would be expected due to a part-whole relationship. In the foundational paper that introduced the spinning fineness equation, genetic correlations between SpinF and MFD ranged from 0.93 to 0.98, depending on the test dataset (Butler and Dolling 1992). Originally, these authors proposed SpinF as a characteristic of spinning performance to be used as an alternative to MFD. Given the strong genetic correlation between MFD and SpinF, only one of these traits needs to be considered in selection decisions. However, SpinF had weak genetic and phenotypic correlations with CVD at −0.15 ± 0.18 and 0.08, respectively. CVD of diameter has a minor contribution in the SpinF equation, where its influence is only through an adjustment factor, which could be why the relationship is weak.

The only phenotypic correlation involving MFD that was not significantly different from zero was with SL (0.06). The genetic correlation between MFD and SL was 0.34 ± 0.13. A much smaller genetic correlation of 0.09 ± 0.01 between MFD and SL was reported for Inner Mongolian White cashmere goats (Bai et al 2005). Down fiber length is a more commonly reported trait in cashmere production compared to staple length but are not the same trait and cannot be compared. Additionally, there is not a standardized definition or measurement method for length traits, which makes direct comparisons difficult.

MFD had a positive phenotypic correlation with FW (0.38), and very similar genetic correlation of 0.40 ± 0.22. Although FW sample size was small and standard errors were large, the moderate, positive genetic correlation suggests that selection for lower MFD would also lead to lighter FW, which is unfavorable. A very similar genetic correlations of 0.48 ± 0.11 was reported for feral Australian goats (Couchman and Wilkinson 1987). A higher genetic correlation of 0.69 ± 0.13 was reported for New Zealand goats (Bigham et al 1993). Two studies reported low genetic correlations between MFD and FW of 0.12 ± 0.23 and 0.13 ± 0.30, although these estimates were also associated with high standard errors (Gifford et al 1990 and Baker et al 1991, respectively). A future study with a large FW sample size is needed to better understand the relationship between MFD and FW. Since cashmere producers are paid based on clean down weight, it would potentially be more useful to evaluate that trait in the future as well.

The correlations between MFD and Perc_19 were very strongly negative, with a genetic correlation of −0.95 ± 0.02 and phenotypic correlation of −0.94. This means as MFD becomes finer, the percentage of fibers with diameter ≤ 19 µm increases, which is a logical relationship. Given this extremely strong genetic correlation, Perc_19 does not appear to characterize additional information beyond what is already explained by MFD. Including both MFD and Perc_19 in selection decisions is likely unnecessary. Because Perc_19 is a novel cashmere trait, there are no correlations published in literature for comparison.

#### Correlations between other fiber characteristics

The phenotypic correlation between CURV and SpinF was −0.66 and genetic correlation was −0.71 ± 0.07. This suggests that increased CURV is associated with a lower SpinF, which indicates finer, higher-quality fiber for spinning. This aligns with results from McGregor and Butler (2008a) which reported that greater fiber CURV is associated with more efficient processing, particularly dehairing. This is similar to the relationship between CURV and MFD, where increased CURV was associated with finer MFD, which is logical because SpinF is calculated largely based on MFD.

CURV had a negative phenotypic correlation of −0.41 with SDD and a similar genetic correlation of −0.42 ± 0.16. This correlation suggests that selection for decreased SDD, or more uniform fiber diameter, would be associated with an increase in CURV. This may be favorable if the breeding objective is to increase CURV, although this will depend on the individual producer and their textile processor’s requirements. McGregor and Butler (2009) reported the same directionality for the relationship between CURV and SDD, where increased SDD was associated with a small decline in CURV. Phenotypically, as SDD increased from 2.5 to 5.5 µm, CURV decreased slightly from 48 to 46°/mm (McGregor and Butler 2009). The relationship between CURV and the other measure of diameter variation, CVD, was opposite in direction. The genetic and phenotypic correlations between CURV and CVD were 0.21 ± 0.17 and 0.12, respectively. Although the standard error is relatively high, this suggests that selection for decreased CVD would be associated with a decrease in CURV. Overall, the relationship between CURV and fiber diameter variation appears to depend on how variation is defined and its application to selection decisions will depend on individual producers’ breeding goals.

CURV and SDCurv had high phenotypic and genetic correlations of 0.70 and 0.78 ± 0.07, respectively. The positive correlation between CURV and SDCurv means that increased CURV tends to be associated with increased variability in CURV. CURV had negative phenotypic and genetic correlations with SL of −0.16 and −0.57 ± 0.11, respectively. The phenotypic and genetic correlations between CURV and FW were also negative at −0.20 and −0.48 ± 0.25, respectively. These correlations suggest that fiber with greater curvature has a shorter staple length and lighter fleece weighs. This is similar to the relationship between cashmere length and curvature observed by McGregor (2003), where a 10 mm increase in length was associated with a decrease in curvature by 3 to 13.1°/mm, depending on cashmere origin, although this referred to down length rather than SL.

Producers selecting for increased CURV can expect a favorable correlated response in the diameter-related traits of CEM and Perc_19 (Table 4). In contrast, the genetic correlations between CURV and both SL and FW were unfavorable, suggesting that selection for increased CURV would be associated with reduced fiber length and weight. Incorporation of CURV into selection decisions should be done with careful consideration of the desired fiber end-product and its relationships with other fiber traits.

The genetic and phenotypic correlations between SL and other fiber traits were both favorable and unfavorable (Table 4). The relationship greasy FW has with other fiber characteristics is important to consider because greasy FW is often evaluated as an indicator of clean down weight, an economically relevant trait. In this study, the sample size of FW was much smaller compared to other fiber traits, resulting in estimates with relatively large standard errors that should be evaluated with caution. Nonetheless, correlations significantly different from zero involving FW were both favorable and unfavorable (Table 4). However, additional research with a larger FW sample size from many different farms would be necessary to further elucidate the relationship FW has with other fiber traits. Given the presence of favorable and unfavorable genetic correlations among fiber traits, development and application of a multi-trait selection index may be the most effective strategy to manage antagonistic responses and achieve targeted genetic gains.

### Genome-wide association study

**Figure 2:**
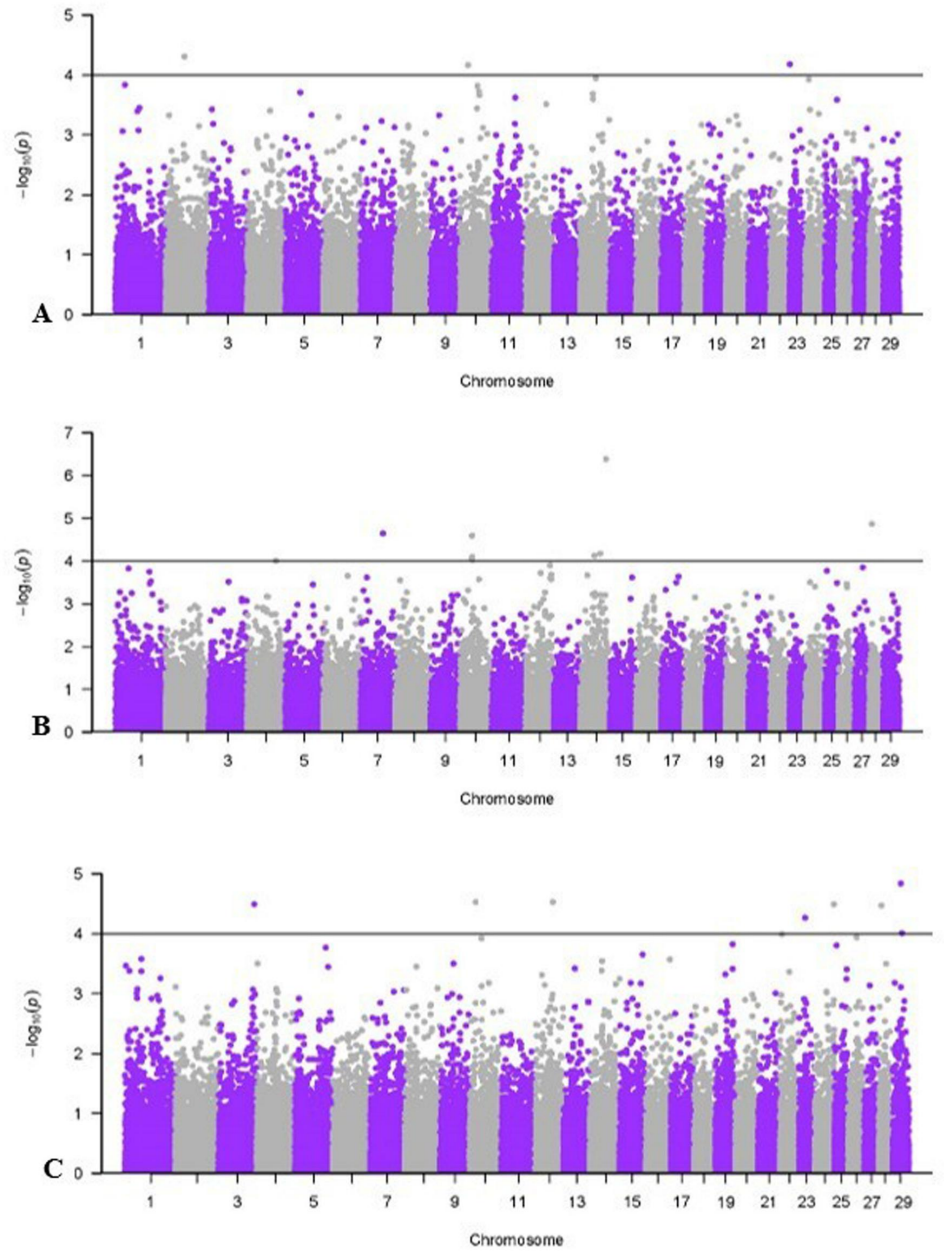
Manhattan plots for univariate, single-record genome-wide association study of mean fiber diameter and diameter variation traits with a significance threshold of 4. A) Mean fiber diameter; B) standard deviation of diameter; C) coefficient of variation of diameter.

**Figure 3:**
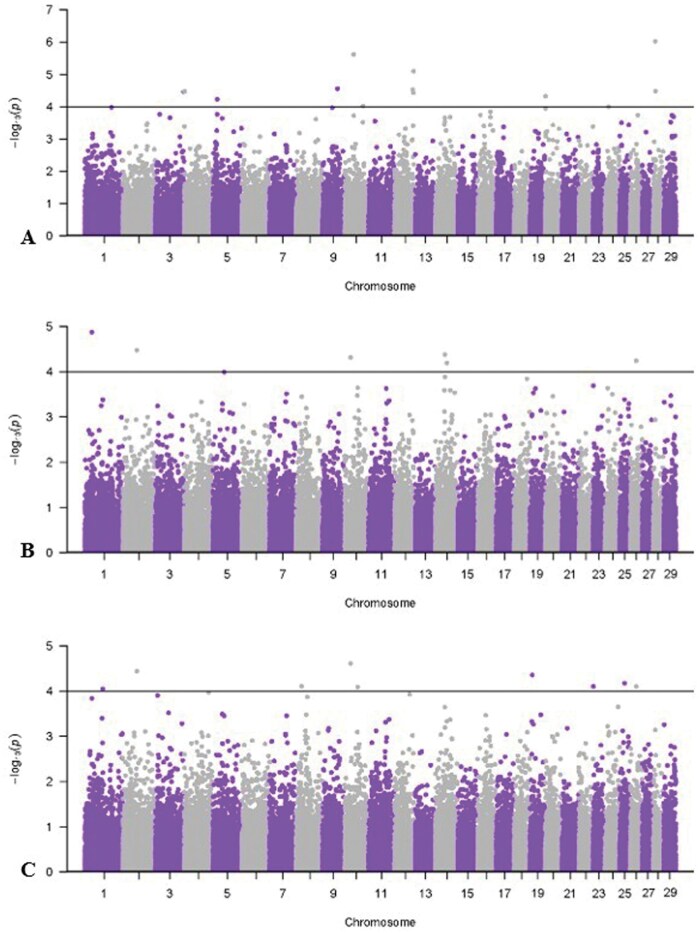
Manhattan plots for univariate, single-record genome-wide association study of traits to describe the fiber diameter distribution with a significance threshold of 4. A) Coarse edge micron; B) spinning fineness; C) percentage of fibers ≤19 µm.

**Figure 4:**
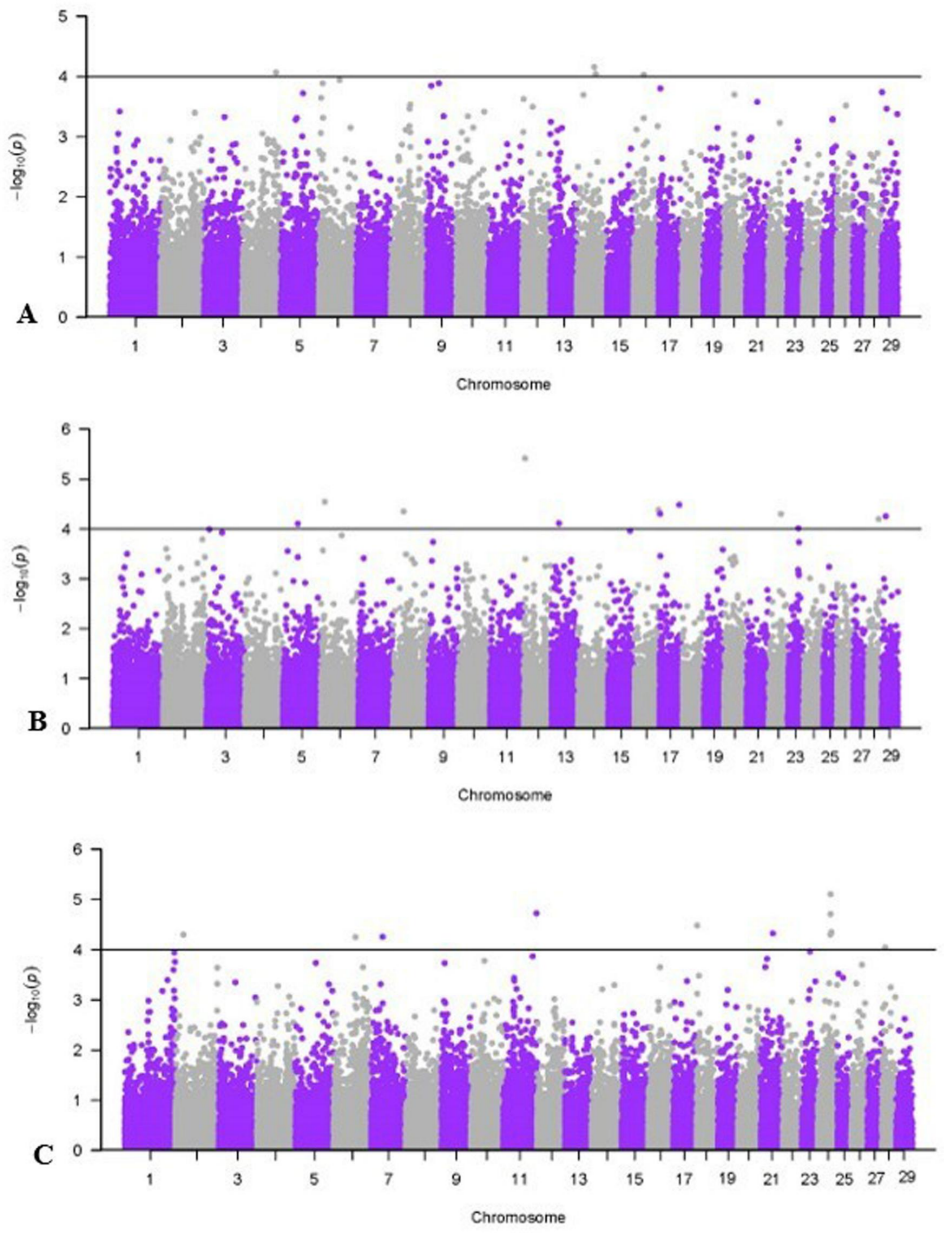
Manhattan plots for univariate, single-record genome-wide association study of curvature and length traits with a significance threshold of 4. A) Curvature; B) standard deviation of curvature; C) staple length.

Table 7 reports significant SNPs for cashmere fiber characteristics, and Manhattan plots are shown in Figures 2–4. All genes within ±50 kilobases of the significant SNP are presented in Table S3. Results for FW GWAS are not presented due to convergence issues from small sample size. A significant threshold of -log_10_  *P*-value ≥ 4 was used to facilitate initial SNP discovery for cashmere fiber traits. The identified associations should be interpreted as exploratory and require validation in future studies.

**Table 7:** Genomic regions significantly^1^ associated with cashmere fiber traits identified through univariate single record genome-wide association study.

Trait	SNP name	Position Chromosome:	-log_10_ *P*-value
**Mean fiber diameter, µm**	snp12723-scaffold1489-359636	2:59355109	4.308
	NC_030830.1_831663	23:831663	4.182
	snp1433-scaffold104-453661	10:25164538	4.167
**Standard deviation of diameter, µm**	14:81337066	14:81337066	6.386
	snp15933-scaffold1674-201551	28:9579785	4.864
	snp46308-scaffold639-126113	7:70168742	4.645
	10:37886722	10:37886722	4.594
	snp13153-scaffold1501-2062871	14:63367161	4.173
	snp15526-scaffold1641-174478	14:45652554	4.126
	snp38104-scaffold475-621550	10:38517075	4.097
	snp38108-scaffold475-788703	10:38683034	4.031
	NC_030811.1_90987564	4:90987564	4.002
**Coefficient of variation of diameter, %**	snp54122-scaffold825-1014475	29:25497494	4.840
	snp9068-scaffold133-715	10:19535805	4.530
	12:54926371	12:54926371	4.529
	snp17668-scaffold183-1269493	3:112157042	4.494
	snp13583-scaffold1525-1372817	24:60678782	4.492
	snp15933-scaffold1674-201551	28:9579785	4.471
	NC_030830.1_18993272	23:18993272	4.266
	snp52988-scaffold796-515837	29:29276909	4.011
**Coarse edge micron, µm**	snp15933-scaffold1674-201551	28:9579785	6.021
	10:37886722	10:37886722	5.613
	snp46521-scaffold644-229314	12:80654586	5.095
	snp43455-scaffold579-4131867	9:62199424	4.550
	snp1151-scaffold1030-421845	12:77016411	4.526
	snp10912-scaffold1390-330847	28:10278379	4.477
	4:2755170	4:2755170	4.466
	NC_030810.1_118553838	3:118553838	4.456
	12:79404524	12:79404524	4.426
	snp52526-scaffold782-3618961	20:2739578	4.319
	snp14293-scaffold157-2174674	5:17950877	4.227
	snp29462-scaffold319-383364	10:77019517	4.012
**Curvature, °/mm**	snp51228-scaffold75-3195161	14:54743414	4.158
	4:105934903	4:105934903	4.071
	snp37050-scaffold449-1431692	14:59014501	4.043
	snp6477-scaffold123-797664	16:33795985	4.030
**Standard deviation of curvature, °/mm**	snp31365-scaffold347-1783379	12:4003713	5.413
	NC_030813.1_11981581	6:11981581	4.545
	snp12495-scaffold1479-395260	17:62391045	4.484
	snp27887-scaffold299-3305456	16:76709383	4.380
	8:33818616	8:33818616	4.349
	snp9919-scaffold1354-42468	17:2617367	4.304
	snp15342-scaffold163-2570163	22:38596654	4.300
	snp48248-scaffold683-100458	29:12983250	4.255
	NC_030835.1_35266324	28:35266324	4.197
	snp22469-scaffold2223-95469	13:22938705	4.115
	snp19615-scaffold1980-634136	5:46781804	4.105
	snp11445-scaffold1417-386228	23:32736234	4.014
**Spinning fineness, µm**	NC_030808.1_29075396	1:29075396	4.873
	snp12723-scaffold1489-359636	2:59355109	4.475
	snp10357-scaffold1370-1004058	14:41058959	4.377
	snp1433-scaffold104-453661	10:25164538	4.313
	snp55570-scaffold861-1053172	26:25703901	4.241
	14:49533115	14:49533115	4.193
**Staple length, mm**	NC_030831.1_40583446	24:40583446	5.103
	11:104347406	11:104347406	4.724
	snp54557-scaffold833-1303845	24:40263321	4.708
	18:2875561	18:2875561	4.481
	NC_030831.1_43366786	24:43366786	4.348
	snp4218-scaffold1130-1285272	21:38165973	4.321
	snp54553-scaffold833-1114417	24:40073220	4.299
	2:26320799	2:26320799	4.297
	snp57722-scaffold93-471433	7:36257418	4.253
	6:68919933	6:68919933	4.247
	NC_030835.1_11100855	28:11100855	4.042
**Percentage ≤19, %**	snp1433-scaffold104-453661	10:25164538	4.611
	snp12723-scaffold1489-359636	2:59355109	4.439
	19:9949651	19:9949651	4.355
	snp36430-scaffold438-690423	25:20239001	4.176
	NC_030815.1_24816118	8:24816118	4.107
	NC_030830.1_831663	23:831663	4.103
	snp55570-scaffold861-1053172	26:25703901	4.101
	snp57298-scaffold912-1705662	10:54284488	4.090
	snp36326-scaffold435-3071866	1:74472653	4.048

1 SNPs (single nucleotide polymorphism) significant at −log_10_  *P*-value of 4.

Three SNPs were significant for MFD, including snp12723-scaffold1489-359636, NC_030830.1_831663, and snp1433-scaffold104-453661 (Table 7). The SNP NC_030830.1_831663 was near two genes: FOXC1 (Forkhead box C1) and GMDS (GDP-mannose 4,6-dehydratase; Table S2). The gene FOXC1 has biological processes that could be related to MFD, making it a possible candidate gene. FOXC1 is involved with the biological process of “collagen fibril organization,” which is a process that determines the size and arrangement of collagen fibrils (The UniProt Consortium 2023). Collagen fibrils are the structural units that make up collagen fibers and are a key component of the extracellular matrix which provides structural support for skin and hair follicles. Proper organization of collagen fibrils is important for follicle development and therefore could influence the diameter of hair grown from secondary hair follicles (cashmere down). In addition, FOXC1 is involved with the biological process of “cellular response to epidermal growth factor (EGF) stimulus” (The UniProt Consortium 2023). This process refers to any change in cell behavior in response to EGF signaling. EGF has a role in hair follicle morphogenesis and the growth phase of the follicle cycle. Altered EGF signaling could affect the activity and size of cells involved with fiber production such as matrix keratinocytes, potentially impacting MFD. The gene FOXC1, is involved with many other functions and processes, but another notable process potentially related to MFD is “positive regulation of keratinocyte differentiation” (The UniProt Consortium 2023). This refers to any biological process that increases the rate, frequency, or extent of keratinocyte differentiation. Keratinocytes are epithelial cells that originate in the basal layer of skin or hair follicles. As they move upward, these cells differentiate and produce different types of keratins at each stage, which is essential for fiber formation. Given the importance of the biological processes of FOXC1 for fiber growth, it is a candidate gene for MFD. While not specifically FOXC1, other members of the Forkhead box gene family, including FOXN1, FOXE1, and FOXI3 have a regulatory effect on the growth and development of hair follicles and the hair growth cycle and were differentially expressed in a study of Inner Mongolia cashmere goats (Han et al 2018).

Nine significant SNPs were identified for SDD (Table 7), and a total of 25 genes were within the QTL regions of these nine SNPs (Table S2). The SNP 14:81337066 was near nine genes, including SCX (Scleraxis basic helix-loop-helix transcription factor). This gene has the biological process of “cellular response to transforming growth factor-beta (TGF-β)” (The UniProt Consortium 2023). Transforming growth factor-beta is a growth factor involved in various cellular processes, including extracellular matrix formation. Signaling for TGF-β plays a role in the differentiation of fibroblasts and keratinocytes in the skin and hair follicle. This is important for hair growth and influences collagen production. Other biological processes related to collagen associated with SCX were “collagen fibril organization” and “positive regulation of collagen biosynthetic process” (The UniProt Consortium 2023). Due to SCX’s involvement in biological processes that influence the formation and structure of fibers, it is a candidate gene for SDD. Another gene near the significant SNP 14:81337066 is DGAT1 (Diacylglycerol O-acyltransferase 1). The DGAT1 gene encodes an enzyme that catalyzes the final and rate-limited step of triglyceride synthesis, playing a central role in lipid metabolism. Due to its role in lipid metabolism, DGAT1 could impact skin and sebaceous gland function, potentially affecting the skin lipid barrier and follicular environment. While not directly related to fiber characteristics, the DGAT1 gene was previously associated with 11 different traits in sheep, including: body weight, hot carcass weight, longissimus muscle area, shoulder weight, and subcutaneous fat thickness (Armstrong et al 2018); leg yield, loin yield, and shoulder yield (Dai et al 2022); and milk fat percentage, milk protein percentage, and milk yield (Scata et al 2009).

For CVD, eight SNPs were significant (Table 7). There were 24 genes within the QTL regions (Table S2). Of the 24 genes, 7 were uncharacterized and 6 were variants of the histone gene family. The SNP 12:54926371 was near the gene FLT1 (fms-related receptor tyrosine kinase 1), which encodes a receptor tyrosine kinase involved in receptor binding of several growth factors, including EGF and fibroblast growth factor (FGF). Both these ligands are highly involved in skin and follicle development. The role of EGF in follicle morphogenesis and cycling was discussed previously for FOXC1, a candidate gene for MFD, as it is also involved with cellular response to EGF stimulus. FGFs are involved in a wide range of biological processes but play a critical role in hair follicle development and cycling. For example, FGFs regulate hair follicle induction during embryonic development. They also signal the transition from the anagen (growth) to the catagen (regression) phase of the hair cycle, promote proliferation of dermal papilla cells, and interact with keratinocytes. In cashmere goats, different members of the FGF gene family have been associated with secondary hair follicle development (He et al 2016; Daio et al 2023; Gao et al 2023). These connections support FLT1 as a candidate gene for fiber diameter variation, or CVD.

Twelve SNPs were significant for CEM (Table 7). The SNP 10:37886722 was significant for both CEM and SDD. The QTL region of this SNP contained six genes, including SORD (sorbitol dehydrogenase), DUOX2 (dual oxidase 2), DUOXA2 (dual oxidase maturation factor 2), DUOXA1 (dual oxidase maturation factor 1), DUOX1 (dual oxidase 1), and SHF (SH2 domain-containing protein). These genes are broadly involved in oxidative stress regulation, epithelial cell function, and various signaling pathways. Specifically, SORD encodes an enzyme involved in sorbitol metabolism and redox balance. The DUOX gene family produces hydrogen peroxide in epithelial tissues and plays a role in mucosal immunity. SHF is an adaptor protein involved with intracellular signaling, including growth factor pathways. Although their direct connection to fiber development is unclear, their functions in oxidative stress response and epithelial signaling could impact the follicle environment and, in turn, fiber characteristics like CEM. Three genes near significant SNPs for CEM, IL22RA2 (interleukin-22 receptor subunit alpha-2), IFNGR1 (interferon gamma receptor 1), and CGN (collectin-46), are involved in immune and inflammatory pathways. The gene IL22RA2 encodes a receptor that modulates interleukin-22 (IL-22) signaling, which is important for epithelial cell repair, proliferation, and immune response in the skin. In humans, it has been reported that IL-22 induces keratinocyte proliferation and inhibits keratinocyte differentiation in connection to psoriasis, a skin disease characterized by hyperproliferation of keratinocytes and infiltration of inflammatory cells (Zhuang et al 2020). The influence that IL-22 has on keratinocyte behavior makes IL22RA2 a gene candidate for CEM. Another gene, CGN, is involved in the innate immune system and binds to carbohydrate patterns on pathogens to initiate immune response. Additionally, CGN is associated with the cellular component “collagen trimer” (The UniProt Consortium 2023), which is the basic structural unit of collagen, giving it its strength and stability. Thus, CGN is another candidate gene for CEM due to its potential roles in immune response or extracellular matrix interactions that could influence the follicular environment.

Curvature had four significant SNPs above the -log_10_  *P*-value threshold of 4 (Table 7). Four genes were within the QTL regions of significant SNPs for CURV, including TGS1 (trimethylguanosine synthase 1), TMEM68 (transmembrane protein 68), KMO (kynurenine 3-monooxygenase), and FH (fumarate hydratase). The gene TMEM68 was near snp37050-scaffold449-1431692 and is involved in lipid metabolism, specifically the biosynthesis of triglycerides. Alterations in lipid metabolism could influence the lipid composition of cell membranes in hair follicles, affecting hair follicle function. The gene KMO was near snp6477-scaffold123-797664. It is involved in the kynurenine pathway and is associated with immune response and oxidative stress, which could potentially impact hair follicle health and activity. While these genes do not have direct implications for fiber curvature, TMEM68 and KMO are plausible gene candidates due to their roles in lipid metabolism and immune response, respectively.

Standard deviation of curvature had 12 significant SNPs (Table 7). The most notable gene within the QTL regions of these significant SNPs was EFNB2 (ephrin-B2). This gene is important for various developmental processes and expressed in the skin during hair follicle development. One of EFNB2’s biological processes is “negative regulation of keratinocyte proliferation” (The UniProt Consortium 2023). Since keratinocytes are the predominant cell type in the epidermis and outer root sheath of hair follicles, this biological process is particularly relevant in the context of a fiber trait such as SDCurv. In mice, the influence EFNB2 has on hair follicle biology has been studied. Negative regulation of cell proliferation in the epidermis and hair follicle was observed in vivo in mice where disruption of ephrin receptor interactions was associated with increased cell proliferation (Genander et al 2010). Additionally, it has been reported that EFNB2 is transiently expressed in hair buds during embryogenesis and in dermal mesenchymal cells during the perinatal period (Egawa et al 2009), suggesting a role in hair follicle morphogenesis. The influence EFNB2 has on keratinocyte cell behavior makes it a reasonable candidate gene for SDCurv. The gene NEFH (neurofilament heavy polypeptide) was near snp9919-scaffold1354-42468. This gene encodes heavy neurofilament chains, which help maintain neuron structure. Although NEFH is primarily expressed in nervous tissue and not the skin or hair follicles, it was previously associated with fleece yield in Chinese fine-wool sheep (Zhao et al 2021), suggesting a potential indirect or regulatory role in fiber production that warrants further research.

Six SNPs were significant for SpinF (Table 7). Of those six SNPs, only three had genes within their QTL region (Table S2). Two genes were uncharacterized, and the gene CNTNAP5 (contactin-associated protein-like 5) was near snp12723-scaffold1489-359636. This gene is a member of the neurexin family and is involved in nervous system development and function through cell adhesion and intercellular communication. More research would be needed to elucidate whether CNTNAP5 could be functionally linked to SpinF and a plausible candidate gene.

Staple length had 11 SNPs above the -log_10_  *P*-value threshold of 4 (Table 7). Thirteen genes were identified within the QTL regions of these SNPs (Table S2). The EPHA4 (EPH receptor A4) gene encodes a receptor in the Eph family of receptor tyrosine kinases. It is directly connected to EFNB2, previously discussed with SDCurv, as both genes are members of the ephrin signaling pathway. Gene Ontology molecular functions for EPHA4 include “EGF receptor activity,” “FGF receptor activity,” and “protein tyrosine kinase collagen receptor activity” (The UniProt Consortium 2023), which are all relevant to skin and hair follicle development. Specifically, FGF signaling influences hair follicle morphogenesis and anagen phase initiation, which could impact SL. Additionally, EPNA4 has an influence on collagen and extracellular matrix interactions, which could affect tissue structure and thus SL. The identification of both EPHA4 and EFNB2, genes within the same signaling pathway near significant SNPs in this analysis suggests that ephrin signaling is connected to cashmere fiber production.

Nine SNPs were significant for Perc_19 (Table 7) and 11 genes were in the QTL regions of these SNPs (Table S2). One SNP, NC_030830.1_831663, was common between Perc_19 and MFD. This SNP was near the gene FOXC1, which has obvious connections to fiber production, as discussed previously. It is logical that Perc_19 and MFD would share significant SNPs, as these two traits are highly genetically correlated. Another SNP, snp57298-scaffold912-1705662, was near the gene RORA (retinoic acid-related orphan receptor A). This gene encodes a nuclear receptor involved in a range of biological processes, including circadian rhythm, metabolism, and immune response. It has been reported that RORA regulates autophagy of hair follicle stem cells (HFSCs) and that increased autophagy of HFSCs promotes the start of the anagen phase of the hair cycle (Zhao et al 2025). Additionally, RORA has been connected to melatonin signaling and seasonal hair molting, where daylight changes influence melatonin levels, thus affecting RORA activity and the hair growth cycle (Zhang et al 2025). Given its roles in the timing of the hair growth cycle and HFSC regulation, RORA is a strong candidate gene for Perc_19. Lastly, the gene fibroblast growth factor 12 (FGF12) was in the QTL region for snp36326-scaffold435-307186. This gene encodes a member of the FGF family, which has known roles in cell growth, tissue repair, and development. FGF12 is primarily expressed in the outer root sheath cells of hair follicles and has high expression during the anagen phase of the hair cycle (Woo et al 2022). In mice, knockdown of FGF12 delayed telogen to anagen phase transition, further supporting that FGF12 is associated with hair growth promotion (Woo et al 2022). Further, Wang et al (2021) identified FGF12 as a candidate gene for fiber length in a GWAS on Inner Mongolia cashmere goats. These findings suggest FGF12 is a reasonable candidate gene for cashmere fiber traits like Perc_19.

## Conclusion

Literature on the genetic control of cashmere production is limited, especially in the U.S. Producers in the U.S. lack a genetic selection tool to inform their breeding decisions. Development and implementation of a national genetic evaluation would aid producers in improving their cashmere flocks, leading to increased production, profitability, and efficiency. In general, fiber characteristics are moderate to highly heritable. Most fiber characteristics have high repeatability, suggesting that the first fiber record tested on an animal is a good indicator of subsequent records, and animals likely only need their fiber tested once in their lifetime, not every year. Phenotypic and genetic correlations varied depending upon the pair of traits. Genetic correlations between MFD and other traits were favorable for SDD, CEM, CURV, SpinF, and Perc_19. However, genetic antagonisms between MFD and CVD, SDCURV, SL, and FW need to be considered when placing heavy selection emphasis on reduced MFD. Therefore, a selection index that properly weights each trait may be beneficial for cashmere producers. The genome-wide association study identified many significant SNPs that were in proximity to genes previously implicated in fiber production or related biological processes. Notable candidate genes include FOXC1, SCX, FLT1, EFNB2, EPHA4, RORA, and FGF12. These results provide initial estimates of genetic parameters for cashmere fiber traits in U.S. goats, where limited information is currently available. Additional research using expanded datasets is warranted to further refine and strengthen these findings prior to the development of genetic selection tools for cashmere production in the United States.

## Supplementary Material

skag073_Supplementary_Data

## Abbreviations

AIC: Akaike information criterion
CEM: coarse edge micron
CURV: curvature
CVD: coefficient of variation of mean fiber diameter
EGF: epidermal growth factor
FGF: fibroblast growth factor
FW: fleece weight
GWAS: genome wide association study
HFSC: hair follicle stem cells
IL-22: interleukin-22
MFD: mean fiber diameter
OFDA: optical fibre diameter analyser
Perc_19: percentage less than equal to 19 µm
QTL: quantitative trait loci
SDCurv: standard deviation of fiber curvature
SDD: standard deviation of fiber diameter
SL: staple length
SNP: single nucleotide polymorphism
SpinF: spinning fineness
TGF-β: transforming growth factor-beta
