# Supplementary material for: Genetic parameter estimation and genome-wide association study of fiber characteristics for cashmere goats in the United States
Source: J Anim Sci. 2026 Mar 13;104:skag073. doi: 10.1093/jas/skag073 (PMC13049200; doi:10.1093/jas/skag073)
Supplement: skag073_Supplementary_Data [file skag073_supplementary_data.docx]

**Supplementary Tables**

Table S1. Genetic covariances between fiber characteristics from bivariate analyses.

|  | MFD | SDD | CVD | CEM | CURV | SDCurv | SpinF | SL | FW | Perc_19 |
| --- | --- | --- | --- | --- | --- | --- | --- | --- | --- | --- |
| MFD, µm |  | 0.1176 | -0.4424 | 0.3143 | -4.904 | -2.105 | 0.8309 | 3.4928 | 22.585 | -6.1779 |
| SDD, µm |  |  | 0.2184 | 0.1598 | -0.6712 | -0.1860 | 0.1424 | 0.7544 | 0.0863 | -0.9345 |
| CVD, % |  |  |  | 0.4974 | 2.0614 | 1.5017 | -0.1754 | 1.2069 | -24.95 | 2.3779 |
| CEM, µm |  |  |  |  | -1.769 | -0.3452 | 0.3814 | 2.1777 | 1.7813 | -2.3703 |
| CURV, º/mm |  |  |  |  |  | 20.174 | -4.4062 | -47.310 | -190.36 | 32.69 |
| SDCurv, º/mm |  |  |  |  |  |  | -1.7922 | -26.156 | -109.13 | 13.738 |
| SpinF, µm |  |  |  |  |  |  |  | 3.3794 | 19.376 | -5.9148 |
| SL, mm |  |  |  |  |  |  |  |  | 371.62 | -21.859 |
| FW, g |  |  |  |  |  |  |  |  |  | -255.52 |
| Perc_19, % |  |  |  |  |  |  |  |  |  |  |

MFD- mean fiber diameter; SDD- standard deviation of fiber diameter; CVD- coefficient of variation of fiber diameter; CEM- coarse edge micron; CURV- curvature; SDCurv- curvature standard deviation; SpinF- spinning fineness; SL- staple length; FW-fleece weight; Perc_19- percentage of fibers with diameter less than or equal 19 µm.

Table S2. Gene candidates in the linkage disequilibrium range (± 50 kb)^1^ associated with each significant^2^ single nucleotide polymorphism (SNP) for each fiber trait.

| Trait | | SNP name | | Chromosome: Position | Gene abbreviation | Full gene name |
| --- | --- | --- | --- | --- | --- | --- |
| Mean fiber diameter | | NC_030830.1_831663 | | 23:831663 | FOXC1 | Forkhead box C1 |
|  |  |  |  |  | GMDS | GDP-mannose 4,6-dehydratase |
|  |  | snp12723-scaffold1489-359636 | | 2:59355109 | CNTNAP5 | Contactin-associated protein-like 5 |
|  |  | snp1433-scaffold104-453661 | | 10:25164538 | LOC108636956 | Uncharacterized |
| Standard deviation of diameter | | snp15526-scaffold1641-174478 | | 14:45652554 | SBSPON | Somatomedin B and thrombospondin type 1 domain containing |
|  |  |  |  |  | TERF1 | Telomeric repeat-binding factor |
|  |  | 14:81337066 | | 14:81337066 | MROH1 | Maestro heat like repeat family member 1 |
|  |  |  |  |  | BOP1 | Ribosome biogenesis protein |
|  |  |  |  |  | SCX | Basic helix-loop-helix transcription factor scleraxis |
|  |  |  |  |  | HSF1 | Heat shock transcription factor 1 |
|  |  |  |  |  | DGAT1 | Diacylglycerol O-acyltransferase |
|  |  |  |  |  | SCRT1 | Scratch family transcriptional repressor 1 |
|  |  |  |  |  | TMEM249 | Transmembrane protein 249 |
|  |  |  |  |  | FBXL6 | F-box/LRR-repeat protein 6 isoform X1 |
|  |  |  |  |  | SLC52A2 | Riboflavin transporter |
|  |  |  |  |  | ADCK5 | AarF domain-containing protein kinase 5 isoform X2 |
|  |  |  |  |  | CPSF1 | Cleavage and polyadenylation specificity factor subunit 1 isoform X2 |
|  |  | snp46308-scaffold639-126113 | | 7:70168742 | LOC102183958 | IRG-type G domain-containing protein |
|  |  |  |  |  | LOC102171703 | Ig-like domain-containing protein |
|  |  |  |  |  | TRIM52 | Small ribosomal subunit protein RACK1 |
|  |  |  |  |  | RACK1 | Small ribosomal subunit protein RACK1 |
|  |  |  |  |  | TRIM41 | E3 ubiquitin-protein ligase TRIM41 isoform X1 |
|  |  | 10:37886722 | | 10:37886722 | SORD | Sorbitol dehydrogenase |
|  |  |  |  |  | DUOX2 | Dual oxidase 2 |
|  |  |  |  |  | DUOXA2 | Dual oxidase maturation factor 2 |
|  |  |  |  |  | DUOXA1 | Dual oxidase maturation factor 1 |
|  |  |  |  |  | DUOX1 | Dual oxidase 1 |
|  |  |  |  |  | SHF | SH2 domain-containing protein |
|  |  | NC_030811.1_90987564 | | 4:90987564 | SP8 | Transcription factor Sp8 |
| Coefficient of variation of diameter | snp9068-scaffold133-715 | | | 10:19535805 | RGS6 | Regulator of G protein signaling 6 |
|  | 12:54926371 | | | 12:54926371 | PAN3 | PAN2-PAN3 deadenylation complex subunit |
|  |  |  |  |  | FLT1 | fms related receptor tyrosine kinase 1 |
|  | snp17668-scaffold183-1269493 | | | 3:112157042 | TRNAL-CAG | Uncharacterized |
|  |  |  |  |  | TRNAG-UCC | Uncharacterized |
|  |  |  |  |  | TRNAD-GUC | Uncharacterized |
|  |  |  |  |  | TRNAN-GUU | Uncharacterized |
|  |  |  |  |  | LOC102185698 | Low affinity immunoglobulin gamma Fc region receptor III-A |
|  |  |  |  |  | LOC108635605 | Endogenous retrovirus group K member 6 Gag polyprotein-like |
|  | snp54122-scaffold825-1014475 | | | 29:25497494 | TMEM86A | lysoplasmalogenase |
|  |  |  |  |  | SPTY2D1 | Protein SPT2 homolog |
|  |  |  |  |  | LOC106503731 | Uncharacterized |
|  |  |  |  |  | UEVLD | Ubiquitin-conjugating enzyme E2 variant 3 isoform X3 |
|  | snp13583-scaffold1525-1372817 | | | 24:60678782 | PIGN | GPI ethanolamine phosphate transferase 1 |
|  | NC_030830.1_18993272 | | | 23:18993272 | LOC108633414 | Histone H2B |
|  |  |  |  |  | LOC102178586 | Histone H2A |
|  |  |  |  |  | LOC102178319 | Histone H3 |
|  |  |  |  |  | LOC102180307 | Histone H4 |
|  |  |  |  |  | LOC102178901 | Histone H3.1-like |
|  |  |  |  |  | LOC106501755 | H1.5 linker histone, cluster member |
|  |  |  |  |  | TRNAM-CAU | Uncharacterized |
|  |  |  |  |  | TRNAG-GCC | Uncharacterized |
|  |  |  |  |  | LOC102181115 | Olfactory receptor |
|  | snp52988-scaffold796-515837 | | | 29:29276909 | KIRREL3 | Kirre like nephrin family adhesion molecule 3 |
| Course edge micron | snp10912-scaffold1390-330847 | | | 28:10278379 | CGN | Collectin-46 |
|  | snp52526-scaffold782-3618961 | | | 20:2739578 | KCNIP1 | Potassium voltage-gated channel interacting protein 1 |
|  |  |  |  |  | GABRP | Gamma-aminobutyric acid receptor subunit pi |
|  | 4:2755170 | | | 4:2755170 | LOC106502073 | uncharacterized |
|  |  |  |  |  | INSIG1 | Insulin-induced gene protein |
|  | 10:37886722 | | | 10:37886722 | SORD | Sorbitol dehydrogenase |
|  |  |  |  |  | DUOX2 | Dual oxidase 2 |
|  |  |  |  |  | DUOXA2 | Dual oxidase maturation factor 2 |
|  |  |  |  |  | DUOXA1 | Dual oxidase maturation factor 1 |
|  |  |  |  |  | DUOX1 | Dual oxidase 1 |
|  |  |  |  |  | SHF | SH2 domain-containing protein |
|  | snp29462-scaffold319-383364 | | | 10:77019517 | LOC102169186 | Olfactory receptor |
|  |  |  |  |  | HNRNPC | Heterogeneous nuclear ribonucleoproteins C1/C2 isoform X2 |
|  |  |  |  |  | RPGRIP1 | RPGR interacting protein 1 |
|  | 12:79404524 | | | 12:79404524 | LOC108637264 | Germinal center-associated signaling and motility-like protein |
|  | snp43455-scaffold579-4131867 | | | 9:62199424 | LOC108636733 | uncharacterized |
|  |  |  |  |  | IL22RA2 | Interleukin-22 receptor subunit alpha-2 |
|  |  |  |  |  | IFNGR1 | Interferon gamma receptor 1 |
| Curvature | | | snp37050-scaffold449-1431692 | 14:59014501 | TGS1 | Trimethylguanosine synthase 1 |
|  |  |  |  |  | TMEM68 | Transmembrane protein 68 |
|  |  |  | snp6477-scaffold123-797664 | 16:33795985 | KMO | Kynurenine 3-monooxygenase |
|  |  |  |  |  | FH | Fumarate hydratase, mitochondrial |
| Standard deviation of curvature | | | snp9919-scaffold1354-42468 | 17:2617367 | NEFH | Neurofilament heavy polypeptide |
|  |  |  |  |  | LOC106503044 | Uncharacterized |
|  |  |  |  |  | AP1B1 | AP complex subunit beta |
|  |  |  |  |  | RASL10A | RAS like family 10 member A |
|  |  |  | snp31365-scaffold347-1783379 | 12:4003713 | ARGLU1 | Arginine and glutamate rich 1 |
|  |  |  |  |  | EFNB2 | Ephrin-B2 |
|  |  |  | NC_030813.1_11981581 | 6:11981581 | LOC108636202 | Uncharacterized |
|  |  |  |  |  | ARSJ | Arylsulfatase J |
|  |  |  | snp22469-scaffold2223-95469 | 13:22938705 | SPAG6 | Sperm-associated antigen 6 |
|  |  |  | 11:73305234 | 11:73305234 | ASXL2 | Polycomb group protein ASXL2 isoform X1 |
|  |  |  | snp11445-scaffold1417-386228 | 23:32736234 | TRERF1 | Transcriptional-regulating factor 1 isoform X2 |
| Spinning fineness | | | snp12723-scaffold1489-359636 | 2:59355109 | CNTNAP5 | Contactin-associated protein-like 5 |
|  |  |  | 14:49533115 | 14:49533115 | C14H8orf34 | Uncharacterized |
|  |  |  | snp1433-scaffold104-453661 | 10:25164538 | LOC108636956 | Uncharacterized |
| Staple length | | | snp54557-scaffold833-1303845 | 24:40263321 | ARHGAP28 | Rho GTPase activating protein 28 |
|  |  |  |  |  | LAMA1 | Laminin subunit alpha-1 isoform X1 |
|  |  |  | NC_030831.1_40583446 | 24:40583446 | SPIRE1 | Protein spire homolog 1 isoform X2 |
|  |  |  |  |  | CEP76 | Centrosomal protein of 76 kDa |
|  |  |  |  |  | PSMG2 | Proteasome assembly chaperone 2 |
|  |  |  | 11:104347406 | 11:104347406 | LOC108637096 | Uncharacterized |
|  |  |  | 18:2875561 | 18:2875561 | SF3B3 | Splicing factor 3B subunit 3 |
|  |  |  |  |  | COG4 | Conserved oligomeric Golgi complex subunit 4 |
|  |  |  |  |  | FUK | L-fucose kinase isoform X1 |
|  |  |  | 2:26320799 | 2:26320799 | EPHA4 | EPH receptor A4 |
|  |  |  | 6:68919933 | 6:68919933 | USP46 | Ubiquitin carboxyl-terminal hydrolase 46 |
|  |  |  |  |  | LOC108636224 | Uncharacterized |
|  |  |  |  |  | LOC102170130 | Uncharacterized |
| Percentage ≤ 19µm | | | NC_030815.1_24816118 | 8:24816118 | ACER2 | Alkaline ceramidase |
|  |  |  |  |  | RPS6 | 40S ribosomal protein S6 |
|  |  |  |  |  | DENND4C | DENN domain containing 4C |
|  |  |  | NC_030830.1_831663 | 23:831663 | FOXC1 | Forkhead box C1 |
|  |  |  |  |  | GMDS | GDP-mannose 4,6-dehydratase |
|  |  |  | snp1433-scaffold104-453661 | 10:25164538 | LOC108636956 | Uncharacterized |
|  |  |  | snp57298-scaffold912-1705662 | 10:54284488 | RORA | Retinoic acid-related orphan receptor A |
|  |  |  | snp12723-scaffold1489-359636 | 2:59355109 | CNTNAP5 | Contactin-associated protein-like 5 |
|  |  |  | 19:9949651 | 19:9949651 | YPEL2 | Protein yippee-like 2 isoform X2 |
|  |  |  | snp36326-scaffold435-3071866 | 1:74472653 | FGF12 | Fibroblast growth factor 12 |
|  |  |  |  |  | LOC106502093 | Uncharacterized |

^1^If no genes were within the linkage disequilibrium range, then the SNP was not listed in the table.

^2^SNPs significant at -log_10_ *P*-value of 4.
